# Relations between Neurocognitive Function and Visual Acuity: A Cross-Sessional Study in a Cohort of Premature Children

**DOI:** 10.3390/children11080894

**Published:** 2024-07-25

**Authors:** Chun-Hsien Tu, Wei-Chi Wu, Wei-Chih Chin, Shih-Chieh Hsu, I Tang, Jen-Fu Hsu, Hung-Da Chou, Eugene Yu-Chuan Kang, Yu-Shu Huang

**Affiliations:** 1 Department of Psychiatry, New Taipei Municipal Tucheng Hospital, New Taipei City 236, Taiwan; studytu@cgmh.org.tw (C.-H.T.); hsu3160@cgmh.org.tw (S.-C.H.); 2 Department of Psychiatry, Chang Gung Memorial Hospital (Linkou Branch), Taoyuan 33305, Taiwan; kikitang@cgmh.org.tw; 3 Department of Ophthalmology, Chang Gung Memorial Hospital (Linkou Branch), Taoyuan 33305, Taiwan; weichi@cgmh.org.tw (W.-C.W.); hdmorph@cgmh.org.tw (H.-D.C.); yckang@cgmh.org.tw (E.Y.-C.K.); 4 School of Medicine, College of Medicine, Chang Gung University, Taoyuan 33302, Taiwan; auaug0327@cgmh.org.tw (W.-C.C.); hsujanfu@cgmh.org.tw (J.-F.H.); 5 Department of Psychiatry and Sleep Center, Chang Gung Memorial Hospital (Linkou Branch), Taoyuan 33305, Taiwan; 6 School of Medicine, National Tsing Hua University, Hsinchu 300044, Taiwan; 7 Division of Pediatric Neonatology, Department of Pediatrics, Chang Gung Memorial Hospital (Linkou Branch), Taoyuan 33305, Taiwan

**Keywords:** cognitive function, Wechsler Preschool and Primary Scale of Intelligence, vision, retinopathy of prematurity, premature infants

## Abstract

Background: Premature children with retinopathy of prematurity (ROP) have been reported to an have increased risk of visual and neurocognitive impairments, yet little is known about whether vision could affect specific neurocognition. This study aimed to clarify the correlations between neurocognition and vision in premature children. Materials and Methods: This is a nonrandomized, cross-sectional, observational study in a pediatric cohort with five groups: (1) full-term (n = 25), (2) prematurity without ROP (n = 154), (3) prematurity with ROP but without treatment (n = 39), (4) prematurity with ROP and with bevacizumab (IVB) treatment (n = 62), and (5) prematurity with ROP and with laser/laser + IVB treatment (n = 20). Neurocognitive function was evaluated by the Wechsler Preschool and Primary Scale of Intelligence, Fourth Edition (WPPSI-IV) around the age of 4 years. Visual acuity (VA) and refractive errors were tested. Correlations between WPPSI parameters and visual outcomes were analyzed across five groups. Results: Among the 300 recruited children (mean age = 4.02 + 0.97 years, male = 56.3%), 297 were assessed by WPPSI-IV and 142 were assessed by vision tests. The Full-Scale Intelligence Quotient (FSIQ) index was worse in the premature groups. After adjusting for covariates, seven items, including FSIQ-Index (*p* = 0.047), fluid-reasoning index (*p* = 0.004), FR-percentile ranking (*p* = 0.008), object assembly (*p* = 0.034), picture concept (*p* = 0.034), zoo locations (*p* = 0.014) and bug search (*p* = 0.020), showed significant differences between groups. The better the best corrected VA (BCVA), the higher the scores on Verbal Comprehension Index (VCI), VCI-PR, and the subtest of information. Conclusions: Specific cognitive dysfunctions are related to the BCVA in this large cohort. Subtest performance profiles in WPPSI can be affected by prematurity, ROP treatment, and different ROP treatment. FSIQ is generally lower in premature children and even lower in children with ROP.

## 1. Introduction

Following the increased survival of preterm children, previous studies found an increased risk of cognitive impairment later in life and long-term disability in learning, vision, and hearing [1,2,3,4,5]. Previous results of a cohort study revealed that premature infants have a 13.2% higher rate of minor neurodevelopmental dysfunctions at 6 months after birth, and that the earlier premature infants are born, the higher the proportion of minor neurodevelopmental dysfunctions [6]. Studies have also reported that the most common disabilities of premature infants at 2 years of age are developmental and cognitive impairments, which can have a great impact during the school years [7,8]. School-aged preterm-born children have significantly lower cognitive scores, which are directly proportional to the birth weight (BW) and gestational age (GA) [9]. Similarly, young adults born preterm continue to show lower performance in diverse neuropsychological functions [10]. Even the adult intelligence scores for those who were very preterm or with low birth weight are lower than mature controls [11]. The intelligent quotient (IQ) score trajectory in premature infants and children is an important field of investigation and has revealed an increased risk of cognitive impairment later in life.

Thus, it is essential to identify factors affecting both overall and specific cognitive functions in order to enable earlier support and intervention [3,12,13,14]. The visual deficit is an important factor that pervasively affects IQ scores. To date, the relationship between neurocognitive function and vision is poorly understood, as is how visual impairment affects the neurocognitive development of premature infants as they grow up. Studies have suggested that visual and cognitive impairments will continue into adolescence and adulthood, with difficulties in concentration, learning, behavior, emotion, and social adjustment [15]. A recent study with a large sample concluded that age-related differences and changes in near visual acuity (VA) were unlikely to contribute to age-related performance in speech and memory measures [16]. However, these studies applied only a single vision measurement (i.e., near VA) and recruited only healthy participants. Therefore, there may be different findings using more detailed vision measurements and recruiting children with pathological conditions such as retinopathy of prematurity (ROP) who have more apparent sensory-cognition relations [16].

ROP is a vascular proliferative retinal disease with multifactorial etiology in preterm infants. It is the most common ophthalmic condition following preterm birth and is the leading cause of preventable childhood blindness [17,18,19]. Risk factors such as intraventricular hemorrhage (IVH) and prolonged invasive ventilatory support (>7 days) in premature babies may have negative effects on later cognitive functioning [3]. The visual function deficits in ROP children also influence other developmental aspects, such as neurocognitive, psychological, and educational [20]. There is also an increased rate of low IQ in ROP children, as well as preterm individuals without ROP [21,22]. Intravitreal injections of anti-vascular endothelial growth factor (VEGF), such as intravitreal injection of bevacizumab (IVB), a widely used treatment of ROP, are associated with systemic risks since they can enter the systemic circulation and lead to systemic VEGF suppression for up to 8 weeks after injection [23,24,25,26]. Systemic risks include the prevention of VEGF’s role in the normal development of the kidneys, lungs, or brain [27]. However, findings from previous studies regarding IVB’s risk of adverse neurodevelopmental outcomes in ROP children are inconsistent and have several limitations. Some limitations include retrospective study design, different indications of treatment, and the problems of uncooperative patients, resulting in difficulty obtaining an adequate neurodevelopmental assessment [28,29,30,31]. A recent meta-analysis [28] that included eight studies with a total of 700 infants supported previous conclusions that IVB treatment does not increase the risk of neurodevelopmental impairment in ROP children, which is in line with some previous reports [32,33,34]. Namely, the IQ of children with ROP is not affected by IVB treatment per se. Instead, other factors such as comorbid visual and neurocognitive deficits should be considered. Alternative treatments of ROP, other than IVB, may include dexamethasone eye drops before laser ablation, which have been reported to prevent infants with Type 2 ROP from developing Type 1 ROP [35].

ROP can impair all visual functions, including VA, visual fields, contrast sensitivity, and color vision [36,37]. However, these visual deficits could result either from preterm birth, ROP, or brain pathology [36]. Preterm children with or without ROP usually present a complex visual cognitive picture that includes both ocular and non-ocular neurological pathology. The visual cognitive interactions are complex, as evident by so-called cerebral visual impairment (CVI), a neurological disorder caused by deficits of the cerebral visual pathway in the absence of major ocular disease, presenting as typical CVI (with abnormal VA) or “higher-functioning CVI” (with normal or near-normal VA) [38,39]. Moreover, visual cortical dysfunction can even exist in preterm children without retinal or cerebral pathology [40]. Most recently, a new perspective has been proposed: ROP may just be the tip of visuopathy of prematurity (VOP), which includes abnormal vascularization of the retina, alterations of cellular architecture in the retina, choroidal degeneration, and abnormalities in the visual pathway [41].

The role of vision in the neurocognitive function of preterm infants needs further investigation and verification. Currently, there are few studies, and none that detect subtle neurocognitive and visual deficits using more detailed measurements in premature children. Thus, we designed a cross-sectional observational cohort study using the Wechsler Preschool and Primary Scale of Intelligence, Fourth Edition (WPPSI-IV) for neurocognitive assessment and collecting more detailed visual parameters in both term and preterm children without or with ROP. We hypothesized that visual parameters such as VA are risk factors for limitations in later IQ and/or specific neurocognitive functions after preterm infants grow up.

## 2. Materials and Methods

### 2.1. Procedures and Participants

This investigation is part of a broader study [34]. This prospective, nonrandomized, observational cohort study at Chang Gung Memorial Hospital in Taiwan began in April 2015. The hospital Institutional Review Board (No. 201801566A3 and 201801537A3) approved the study. The children’s parent(s) provided written informed consent for enrollment in this study. There were approximately 400-to-500 premature babies in our hospital annually. In this study, there was an established protocol for referring to all cases. All pediatricians and pediatric ophthalmologists in our hospital were informed formally of our study protocol, and all agreed to routinely refer suitable participants. Pediatricians, pediatric psychiatrists, pediatric ophthalmologists, and research assistants worked together to evaluate participants’ birth and physical/clinical status at birth, then their cognitive, developmental, and visual function during the follow-up period. Premature children, with or without ROP, were regularly followed up at our pediatric clinics because of prematurity and related issues. The details of the recruitment and assessment procedures included the following: (1) inviting all parents of preterm babies to sign the informed consent of study before their children were discharged from the hospital, (2) clinical follow-up on the physical, developmental, and visual conditions of the children annually, and (3) arranging WPPSI testing between the ages of 3 and 6 years. Participants in the full-term group were mainly children who were followed up at the ophthalmology department for vision evaluation.

### 2.2. Inclusion and Exclusion Criteria

The inclusion criteria included premature children with or without ROP and full-term children. Prematurity was defined as birth at less than 37 weeks of gestation. The exclusion criteria included children who (1) underwent vitrectomy (defined as a surgical procedure releasing the vitreous traction to the retina to relieve retinal detachment) for Stage 4 or 5 ROP, (2) received anti-VEGF agents other than bevacizumab, (3) had severe neurological deficits such as seizures, severe brain disease, severe ICH, and cerebral palsy, or (4) were unwilling or unable to complete the cognitive assessment. Those who met the inclusion criteria without any exclusion criteria were considered eligible.

### 2.3. Grouping

The final cohort was divided into five groups: Group 1: full term; Group 2: prematurity without ROP; Group 3: prematurity with ROP but without treatment; Group 4: prematurity with ROP and with IVB treatment; and Group 5: prematurity with ROP and with laser/laser + IVB treatment.

### 2.4. Data Collection

Demographic data, neonatal events, health, and ROP data were gathered from the medical records. The demographic data included age, gender, gestational age (GA), birth weight (BW), and Apgar score (i.e., appearance, pulse, grimace, activity, and respiration; a score lower than 7 is a sign that the baby needs medical attention) in the 1st and 5th minutes after birth. Neonatal events and health data included the presence of an atrial septum deficit (i.e., a birth defect of the heart in which there is a hole in the atrial septum), ventricular septum deficit (i.e., a birth defect of the heart in which there is a hole in the ventricular septum), bronchopulmonary dysplasia (BPD) [42], pulmonary hypertension, patent ductus arteriosus (i.e., failure of ductus arteriosus closure within a few days after birth), respiratory distress syndrome, necrotizing enterocolitis [43], IVH [44], periventricular leukomalacia [45,46], anemia, blood transfusion, sepsis, and surfactant use. The severity of ROP was graded according to the Early Treatment for Retinopathy of Prematurity (ETROP) Study [47].

Treatment for ROP was either primary IVB or laser photocoagulation, and the indication for treatment was Type 1 ROP, as defined by the ETROP Study [47]. The risks and benefits of the treatments and the off-label use of bevacizumab were thoroughly explained to the parents. The potential benefits of bevacizumab in ROP treatment include a short procedure, no need for general anesthesia, fewer refractive errors, and fewer visual field defects related to the destruction of the peripheral retina by laser photocoagulation. Potential risks of bevacizumab treatment for ROP include an unknown long-term effect on the neuro-developmental outcome. The parents chose the treatment method and signed an informed consent form.

### 2.5. Ophthalmic Evaluation and Instruments

All enrolled children were invited to take a complete ophthalmic examination that included slit-lamp biomicroscopy, indirect ophthalmoscopy, uncorrected VA, and best-corrected VA (BCVA) assessments. Cycloplegic refraction (spherical power (SPH), cylinder (CYL), and spherical equivalent (SE): SPH + CYL/2) were performed using an automatic kerato-refractometer (KR-8100A; Topcon, Tokyo, Japan), followed by manual refraction to refine the outcome. VA was assessed via Landolt-C optotypes, and the results were converted into logarithms of minimum angle-of-resolution (logMAR) units for statistical analysis. Since BCVA is presented by logMAR values, hence smaller values indicating better vision, a negative correlation *r* value reflects a positive relationship between the BCVA and the subtest score. This same ophthalmic evaluation was also used in similar studies [32,33,34].

### 2.6. Cognitive Function Evaluation

Cognitive abilities were assessed by the Chinese version of the WPPSI-IV (Chinese Behavioral Science Co., Taipei, Taiwan) [48]. This protocol was used in prior published studies [33,34]. The test is divided into two age ranges: the younger band from 2 years, 6 months through 3 years, 11 months (2:6–3:11), and the older band from 4 years, 0 months through 7 years, 7 months (4:0–7:7) [49]. The 2:6–3:11 test has eight subtests that can be combined into three composite primary index scales: (1) Verbal Comprehension Index (VCI) containing subtests of Information (IN) and Receptive Vocabulary, (2) Visual Spatial Index (VSI) containing subtests of Block Design (BD), Object Assembly (OA), and (3) Working Memory Index (WMI) containing subtests of Picture Memory (PM) and Zoo Locations (ZL). These three primary indexes can be further combined to calculate the Full-Scale Intelligence Quotient (FSIQ) score, which reflects the overall intelligence of a tested person. For the 4:0–7:7 test, there are 15 subtests that can be combined into five composite primary index scales: (1) VCI containing subtests of IN and Similarities (SI), (2) VSI containing subtests of BD and OA, (3) WMI containing subtests of PM and ZL, (4) Fluid Reasoning Index (FRI) containing subtests of Matrix Reasoning (MR) and Picture Concepts (PC), and (5) Processing Speed Index (PSI) containing subtests of Bug Search (BS) and Cancellation (CA) [49]. The FSIQ score can also be derived from these five primary indexes. There are various supplemental subtests, in addition to the above-mentioned 6 and 10 subtests used in the younger and older age bands, respectively. Raw scores from the subtests were converted to an age-corrected standard scaled score (M = 10, SD = 3) with scoring software. Specific composite scores can be derived from these scaled scores, including the FSIQ (M = 100, SD = 15).

The original English version of WPPSI-IV was validated by U.S. norms in 1700 children [50]. For the FSIQ, the reliability analysis showed excellent internal consistency with a reliability coefficient of 0.96 and a high test-retest correlation of 0.93 [50]. For the FSIQ of the Chinese version of WPPSI-IV, validation in the Taiwanese population showed an internal consistency reliability coefficient of 0.86–0.96 and a test–retest correlation of 0.72–0.89 [48]. In this study, certified and experienced pediatric psychiatrists and psychologists performed all the assessments, and all were blinded to each patient’s prior ophthalmic history and treatment. The test administration of the WPPSI-IV was critically followed with the established administration procedures [51].

### 2.7. Statistical Analysis

Means and standard deviations were calculated for continuous variables, while frequencies and percentages were calculated for categorical variables. A chi-square test was performed to compare categorical variables. To compare data on the demography, clinical data, WPPSI, and visual parameters between groups were analyzed by ANOVA or ANCOVA, followed by post-hoc analysis. The correlations of cognitive function and visual parameters were analyzed by Pearson’s correlation with Bonferroni correction, which was used for evaluating the interaction effects between groups. We performed a power calculation by software Gpower version 3.1 (input parameters: effect size 0.25, alpha 0.05, power 0.8, and group number 5), which finally resulted in a sample size of 200. The level of significance was set at *p* < 0.05. The effect sizes were calculated as η^2^ for the ANOVA/ANCOVA test, and as Cramer’s V for the chi-square test. In general, η^2^ = 0.01 or Cramer’s V around 0.1 or below indicates a small effect, η^2^ = 0.06 or Cramer’s V around 0.3 indicates a medium effect, and η^2^ = 0.14 or Cramer’s V around 0.5 or higher indicates a large effect. SPSS Version 18 was applied for statistical analyses.

## 3. Results

**Figure 1:**
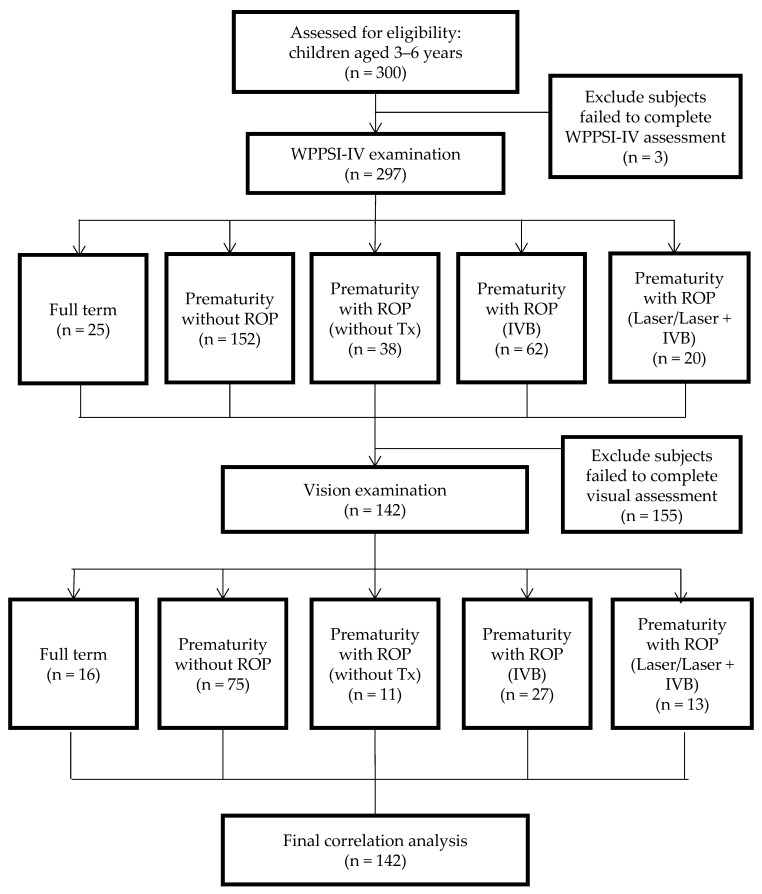
Flow chart of the study protocol (Abbreviations: IVB: intravitreal injection of bevacizumab; ROP: retinopathy of prematurity; Tx: treatment; WPPSI-IV: Wechsler Preschool and Primary Scale of Intelligence, Fourth Edition).

A total of 300 children (mean age = 4.02 + 0.97 years; male = 56.3%) were recruited. Two hundred ninety-seven participants completed the WPPSI-IV examination. Among them, 142 completed vision examinations and were included in the final correlation analysis. The flow chart of this study is shown in Figure 1.

Table 1 shows the demographic and medical information of children at birth. GA, BW, and Apgar scores in 1st and 5th minutes of Groups 1 and 2 were significantly higher than those of other groups (*p* < 0.001) but were not significantly different between Groups 3, 4, and 5 (preterm children with ROP without treatment or with different treatment). Compared with Groups 1 and 2, children in Groups 3, 4, and 5 had higher proportions of BPD, pneumonia, respiratory distress syndrome, patent ductus arteriosus, sepsis, and surfactant use (*p* < 0.001). However, no significant group differences were found between Groups 3, 4, and 5. Incidences of pulmonary hypertension, atrial septum deficit, ventricular septum deficit, necrotizing enterocolitis, IVH, and periventricular leukomalacia also showed no significant difference between ROP Groups 3, 4, and 5. The analysis of ROP stages showed that Groups 4 and 5 had more Stage 3 (i.e., advanced stage of ROP) than Group 3, while Group 3 had more Stage 1 than Groups 4 and 5. In addition, Groups 5 and 4 did not differ significantly in the rates of Stages 1 and 3, whereas Group 5 (and Group 3) had a significantly higher rate of Stage 2 than Group 4. Overall, the neonatal systemic and clinical conditions of Groups 1 and 2 were significantly better than those of the ROP groups (3, 4, and 5).

Table 2 shows the results of the neurocognitive function based on the WPPSI-IV. Three children did not complete the WPPSI-IV, two in group 2 and one in group 3, so data from 297 participants were analyzed. Compared with Groups 1 and 2, the mean FSIQ scores and percentile rankings of Groups 3, 4, and 5 are significantly lower (*p* < 0.001, data not shown). After adjusting for covariates (age, GA, BPD, and IVH) by ANCOVA, seven items, including FSIQ-Index (*p* = 0.047), FRI (*p* = 0.004), FR-Percentile Ranking (*p* = 0.008), OA (*p* = 0.034), PC (*p* = 0.034), ZL (*p* = 0.014), and BS (*p* = 0.020) showed significant differences between the five groups. Through post-hoc correction, we found specific between-group differences in four items: FRI, OA, PC, and BS. For the scores of FRI, Groups 4 and 5 were significantly lower than Groups 1 and 2. For the scores of OA and PC, Group 5 was significantly lower than Groups 1 and 2. For the scores of BS, Groups 4 and 5 were significantly lower than Group 1. No specific group differences were found in FSIQ-I, FR-PR, and ZL by post-hoc correction (Table 2).

One hundred and forty-two children completed the vision examination. Table 3 shows the results of visual parameters such as VA, presented with logMAR values, where smaller values indicate better vision. Group differences are analyzed by ANOVA using Bonferroni’s post-hoc analysis. Group 5 had the worst uncorrected VA (i.e., the largest logMAR value = 0.64 ± 0.42) (*p* < 0.001), and this may relate to the significantly higher spherical power (−1.62 ± 3.16) than Groups 1, 2, 3, and 4. No difference in uncorrected VA and SPH was detected in Groups 1, 2, 3, and 4. After adjusting for age, GA, BPD, and IVH, the findings of visual parameters in these five groups were similar, and Group 5 remained the worst uncorrected VA (*p* = 0.006) and SPH (*p* = 0.003) (data not shown).

Table 4a shows the correlation between WPPSI-IV parameters and the visual outcome. The BCVA of 142 children was correlated to age, GA, BW, FSIQ-I, FSIQ-PR, verbal comprehension-PR, subtests of SI (*r* = −0.231), IN (*r* = −0.278), and BS (*r* = −0.317). SPH was only correlated to age (*r* = −0.230), CYL to ZL (*r* = 0.237), and SE to GA (*r* = −0.012). Table 4b shows the correlation between WPPSI-IV parameters and visual outcomes after adjusting for age, GA, BPD, and IVH. We found that BCVA was correlated to VCI (*r* = −0.192), VC-PR (*r* = −0.286), and subtests of IN (*r* = −0.299), PM (*r* = 0.21), and BS (*r* = −0.228). SPH was correlated to the subtest of SI (*r* = −0.255), whereas CYL was correlated to the subtest of ZL (*r* = 0.301). We had a total of 880 (4 × 22 × 10) tests in the Bonferroni correction for Pearson’s correlation. Finally, only those tests that showed statistical significance are presented in Table S1 (in the Supplement section only), which showed BCVA was significantly correlated to VCI, VCI-PR, IN, BS, and PM.

## 4. Discussion

This study explored correlations between vision and neurocognitive function in preterm children with and without ROP. In this large cohort of full-term and preterm participants who completed the WPPSI-IV (n = 297), we analyzed correlations between vision and neurocognitive functions for five groups of patients (n = 142). The demographic and clinical data of this study show that, as would be expected, the incidence of neonatal complications in premature babies (Groups 2, 3, 4, and 5) is significantly higher than that of full-term babies (Group 1), whereas premature babies without ROP (Group 2) had fewer complications than premature babies with ROP (Groups 3, 4, 5). This indicates a higher morbidity rate in more premature babies, consistent with many previous studies [5,24,32,33,34,52], and it also supports the fact that preventing preterm birth as possible is a very important public health issue.

In addition to health concerns, neurocognitive development is an important issue in preterm children. Previous studies found that the most common and severe developmental impairment in preterm children is cognitive impairment, which was defined as scores less than two standard deviations (SD) below the mean on standardized cognitive measurements, such as Bayley II and III, WPPSI, and WISC [53]. Murray et al. found a higher prevalence of severe cognitive delay in preterm children than full-term children [54]. Their mean IQ is usually in the average or lower average range but significantly lower than their normal birth weight peers [53,55]. A study using the Korean version of WPPSI-IV to assess preterm children’s cognitive function reported that they had poorer cognition than healthy term children at 5 years of age, with mean FSIQ scores of 87 in preterm children and 107 in normal controls [56]. Our WPPSI-IV results had similar findings (Table 2). Most studies of ROP children used the Bayley Scales of Infant Development II or III as the measurement of cognitive functioning between the ages of 6 months and 3 years [25,26,32,33,53]. Our study used the WPPSI-IV to assess cognitive functions and found that preterm ROP groups (groups 3, 4, and 5) had mean IQ scores ranging from 85 to 89, slightly lower than the 0.5 to 1 SD of the mean IQ score 100 in full-term control (Table 2). 

However, although the findings of mean FSIQ in this study showed statistical difference (*p* = 0.047) between groups, the difference became less prominent after adjusting for age, GA, BPD and IVH (Table 2). Interestingly, some domains of WPPSI-IV, including FRI, OA, PC, ZL, and BS, showed significant differences between full-term and preterm children without and with ROP after adjusting for age, GA, BPD and IVH (Table 2). A recent study found that preterm infants who underwent IVB plus laser photocoagulation had a higher rate of severe cognitive impairment at 4.5 years of age [34]. Our study further revealed that premature children with ROP who received laser/laser + IVB (group 5) had not only the worst WPPSI-IV scores but also the worst visual acuity (Table 3). This finding suggests that the development of some cognitive domains can be related to vision. Our findings (Table 2) also revealed that group 5 had the worst performance in FRI and in three subtests including OA, PC, and BS, as compared to that of group 1 (and 2). The groups of treated children (i.e., groups 4 and 5) seemed to have lower FSIQ scores than the other groups after adjusting covariates, though the between-group difference was non-significant. We think the reason may be in large part due to the fact that the treated children were actually those in worse medical (compared to group 1 and 2) and ophthalmological (compared to group 3) conditions.

However, these findings are so distinct and specific that there are difficulties to make comparisons with some previous studies. One study reported that extremely preterm children aged 6.5 years had poorer WMI and perceptual reasoning index of WISC-IV, with the cognitive subscales showing substantial heterogeneity [57]. Another study found that children with very low birth weight (VLBW), defined as BW at birth less than 1500 g, had a global and lasting impact on cognitive ability and their FSIQ score can relate to important learning problems in young adulthood, so VLBW should be assessed to support school and occupational performance [58]. A further study reported that preterm children aged 5.5 had a cognitive pattern marked by weak executive function and that cognitive ability at age 5.5 was highly predictive of that at age 18 [3]. Overall, these previous studies and our recent findings reinforce the importance of cognitive and visual assessments and follow-ups of preterm children, and that these assessments should be initiated early in preschool years.

Preterm birth is associated with an increased risk of cognitive impairment later in life, but the cognitive outcome is heterogeneous. Thus, it is important to identify factors affecting both overall and specific cognitive functions, such as visual deficits, to enable earlier support and intervention [3,12,13,14]. A study of visual deficits in preterm children showed that visual impairments were significantly related to poorer visual perception, motor, and cognitive deficits at school age, which may be caused by a generalized abnormality of cortical development rather than perinatally acquired focal brain lesions [59]. A hypothesis of dorsal stream vulnerability has been proposed for visual deficits and cognitive impairments. It is supported by the finding that preterm birth has a more profound effect on the dorsal visual stream, which is specialized for motion perception and visuomotor control, than the ventral visual stream, which is for form perception [60].

The mechanism and interaction between visual ability and cognition in premature infants and the impact of visual deficits on their development are still uncertain. Regarding the psychological and cognitive evaluation of patients with visual disabilities, there has been very little investigation of cognition and intelligence because of the difficulty working with these patients, with specific needs related to their disability. Evaluation often takes longer and the development of psychological instruments can be even more challenging [61,62]. Since good vision is required for almost all subtests in the WPPSI-IV, except purely verbal subtests, such as IN and SI, poor vision can reduce scores [63]. Our study confirms that the IN and SI subtests were not significantly different between full-term children and children with or without ROP, but there were significant differences in several domains, especially for preterm children with ROP receiving laser/laser + IVB (group 5), who had the worst vision (Table 2). Previous research has concluded that VA examination at 6 years of age can bring lasting benefits for early treatment of ROP, but VA often does not return to its normal development [64]. ROP can have adverse effects on all visual functions, including VA, visual fields, contrast sensitivity, and color vision [36], but differentiating the effects of preterm birth, ROP, and neurological damage can be difficult or even impossible [36]. That preterm children with ROP receiving laser/laser + IVB (group 5) had the worst uncorrected VA may be due to their high rate of neonatal complications, the lowest BW, and the severity of ROP.

After adjusting for covariates (Table 4b), we found BCVA was significantly correlated to VCI, VCI-PR, IN, PM, and BS. However, Table S1 (provided only in the Supplementary section) shows significant between-group interaction effects (*p* < 0.005) between BCVA and only two WPPSI-IV parameters (i.e., BD in the group pair of 1 and 4; BS in the group pairs of 1 and 3, 2 and 3, 3 and 4, 3 and 5). Accordingly, we excluded BS and confirmed that BCVA was indeed correlated with VCI, VCI-PR, IN, and PM. In other words, children with poorer BCVA will have poorer scores in three domains: VCI, VC-PR, and IN. The inverse correlation between BCVA and PM is difficult to interpret. We speculate the following rationale: In addition to the need for BCVA to discriminate the test pictures, other functions, such as attention [65], short-term visual memory [66,67], and visuomotor coordination, were also highly utilized in the PM subtest. In addition, since the PM subtest requires spatial working memory, if ROP children also have reduced visual field despite normal BCVA, they will need to look at the test content part by part and then integrate it through memory, thus hindering their performance. Future research should assess the visual field, as well as higher-level visual-perceptual abilities such as object-recognition difficulties and figure-ground perception, which were not evaluated in this study.

Despite the large sample size, some limitations existed in this study. First, there were fewer children in the laser and the laser-plus-IVB-treatment groups, so they were merged into one group, Group 5. Laser treatment is known to have only local effects, whereas IVB may have a potential systemic anti-VEGF effect that may hinder brain development. Consequently, there could be a possible classification bias by merging the laser and laser-plus-IVB-treatment groups into one group, thus biasing the outcomes in Table 2 and Table 3. Second, only 142 children were able to complete the pediatric clinical vision assessment. The main reasons that a huge number (i.e., 155 in 297) of children did not undergo the visual examination included the long examination time, the discomfort associated with the exams, and the inability of the younger-aged children to cooperate during the testing, as well as the refusal from busy parents, especially in Group 2 (i.e., 77 in 152 children with prematurity without ROP). Parents in this group may feel no urgency to let their children take thorough vision examinations, hence showing noncompliance with the scheduled routine examination. This could potentially lead to a certain selection bias. Third, the study design is cross-sectional, not a longitudinal study, so causality cannot be determined. Fourth, although the WPPSI-IV is a reliable assessment of general cognitive functioning in children aged 4–5 years, it does not consider all neurocognitive functions and does not identify specific learning abilities. Therefore, some subtle cognitive impairments, such as visual-motor skills and learning disability in math, could be missed during preschool age, ones that can often be detected later [53]. These subtle impairments can be highly prevalent and have been reported in 50%-to-70% of children born with very low birth weight [68]. Lastly, we only examined uncorrected VA, BCVA, SPH, CYL, and SE, but other components of visual functions such as contrast sensitivity, visual fields, deficits in eye growth, and eye movements were not measured. Our results should be treated with caution, as this was an exploratory analysis.

Taken together, regular and timely evaluations of vision, as well as cognition for ROP preterm children, should be emphasized to optimize their functional outcomes and adaptation. Future studies on the development of an optimal and clinically practicable vision-cognition screening and assessment are urgently needed. Meanwhile, further understanding of how preterm birth affects vision and cognition, as well as the mechanism between vision and cognitive impairment, is warranted for effective treatments and interventions [15].

## 5. Conclusions

This study confirms specific cognitive patterns in preterm children, especially with ROP who received different treatments for it, and that WPPSI-IV can be a useful tool to assess the neurocognitive function of children born preterm. Specific cognitive functions are affected and related to the best corrected VA. Assessment of the visual field and other higher-level visual perceptual abilities is warranted in future investigation to determine the influence of such abilities on the performance of IQ tests in children with ROP. Early evaluation for both vision and cognition is important in preterm children, and optimal and practical vision–cognition screening assessment is urgently needed. Future long-term studies are required to explore the trajectories of visual and cognitive development.

## Figures and Tables

**Table 1:** Demographic and clinical data (n = 300).

(Mean ± SD)	1. Full-Term (n = 25)	2. Prematurity without ROP (n = 154)	3. Prematurity with ROP (without Tx) (n = 39)	4. Prematurity with ROP (IVB) (n = 62)	5. Prematurity with ROP (Laser/Laser + IVB) (n = 20)	*p* Value	Post Hoc (Scheffe; Bonferroni)	Effect Size ^a^
Age (yrs)	4.06 ± 1.03	3.97 ± 0.98	3.79 ± 0.75	3.98 ± 0.95	4.93 ± 1.30	<0.001 ***	5 > 2,3,4	0.067
Male, n (%)	17 (68.0%)	82 (53.2%)	21 (53.8%)	35 (56.5%)	14 (70.0%)	0.463	n.s.	
GA (wks)	38.33 ± 2.38	31.82 ± 2.98	28.52 ± 2.35	26.83 ± 2.53	25.72 ± 1.49	<0.001 ***	1 > 2 > 3,4,5	0.615
BW (gm)	3162.6 ± 494.11	1635.68 ± 601.44	1074.29 ± 366.28	914.66 ± 302.87	778.67 ± 147.43	<0.001 ***	1 > 2 > 3 > 5 1 > 2 > 4	0.628
Apgar score, 1 min	8.6 ± 0.58	7.3 ± 1.61	4.95 ± 2.04	5.35 ± 2.07	4.45 ± 1.88	<0.001 ***	1 > 2 > 3,4,5	0.345
Apgar score, 5 min	9.6 ± 0.58	8.87 ± 1.25	7.34 ± 1.62	7.44 ± 1.67	6.85 ± 1.46	<0.001 ***	2,1 > 3,4,5	0.281
ROP stage						<0.001 ***		0.626
Stage 1	0 (0%)	0 (0%)	19 (50%)	3 (4.9%)	1 (5.0%)		3 > 1,2,4,5	
Stage 2	0 (0%)	0 (0%)	17 (44.7%)	6 (9.8%)	4 (20.0%)		3,5 > 4 > 2 3 > 1	
Stage 3	0 (0%)	0 (0%)	3 (5.3%)	51 (83.6%)	15 (75.0%)		4,5, > 1,2,3 3 > 2	
Stage 4	0 (0%)	0 (0%)	0 (0%)	0 (0%)	0 (0%)			
Stage 5	0 (0%)	0 (0%)	0 (0%)	1 (1.6%)	0 (0%)			
ASD	1 (4.0%)	28 (18.2%)	6 (15.4%)	19 (30.6%)	8 (40.0%)	0.008 **	5 > 1	0.215
VSD	0 (0.0%)	2 (1.3%)	1 (2.6%)	0 (0.0%)	0 (0.0%)	0.702	n.s.	
BPD	0 (0.0%)	34 (22.1%)	29 (74.4%)	49 (79.0%)	20 (100.0%)	<0.001 ***	3,4,5 > 1,2	0.634
Pneumonia	0 (0.0%)	16 (10.4%)	16 (41.0%)	33 (53.2%)	12 (60.0%)	<0.001 ***	3,4,5 > 1,2	0.481
Pulmonary hypertension	0 (0.0%)	4 (2.6%)	7 (17.9%)	7 (11.3%)	4 (20.0%)	0.001 **	4,5 > 2	0.256
PDA	0 (0.0%)	28 (18.2%)	22 (56.4%)	44 (71.0%)	12 (60.0%)	<0.001 ***	3,4,5 > 1,2	0.519
RDS	0 (0.0%)	88 (57.1%)	37 (94.9%)	62 (100.0%)	20 (100.0%)	<0.001 ***	3,4,5 > 2 > 1	0.619
RDS grade						<0.001 ***		0.370
Grade 1	0 (0.0%)	46 (29.9%)	5 (13.9%)	6 (10.3%)	0 (0.0%)		2 > 1,4	
Grade 2	0 (0.0%)	18 (11.7%)	9 (25.0%)	24 (41.4%)	5 (29.4%)		4 > 1,2; 5 > 1	
Grade 3	0 (0.0%)	18 (11.7%)	8 (22.2%)	14 (24.1%)	8 (47.1%)		5 > 1,2	
Grade 4	0 (0.0%)	7 (4.5%)	11 (30.6%)	13 (22.4%)	4 (23.5%)		3,4,5 > 2; 3 > 1	
NEC	0 (0.0%)	8 (5.2%)	7 (17.9%)	8 (12.9%)	2 (10.0%)	0.030 *	3 > 2	0.200
NEC Stage						0.014 *		0.161
1A	0 (0.0%)	1 (0.6%)	4 (10.3%)	2 (3.2%)	0 (0.0%)		5 > 2	
1B	0 (0.0%)	5 (3.2%)	3 (7.7%)	2 (3.2%)	0 (0.0%)			
2B	0 (0.0%)	1 (0.6%)	0 (0.0%)	2 (3.2%)	0 (0.0%)			
3B	0 (0.0%)	0 (0.0%)	0 (0.0%)	2 (3.2%)	1 (5.3%)			
IVH	0 (0.0%)	26 (16.9%)	14 (35.9%)	19 (30.6%)	8 (40.0%)	<0.001 ***	3,4,5 > 1	0.258
IVH Stage (stage 3 &4)	0 (0.0%)	5 (3.2%)	5 (13.2%)	5 (8.2%)	1 (5.0%)	0.082	n.s.	
PVL	0 (0.0%)	1 (0.6%)	0 (0.0%)	1 (1.6%)	0 (0.0%)	0.844	n.s.	
Anemia	0 (0.0%)	92 (59.7%)	39 (100.0%)	62 (100.0%)	17 (85.0%)	<0.001 ***	3,4 > 2 > 1 4 > 5 > 1	0.610
Blood transfusion	0 (0.0%)	88 (57.1%)	32 (82.1%)	62 (100.0%)	20 (100.0%)	<0.001 ***	4 > 3 > 2 > 1 5 > 2 > 1	0.584
Sepsis	1 (4.0%)	36 (23.4%)	17 (43.6%)	30 (48.4%)	11 (55.0%)	<0.001 ***	4,5 > 1,2; 3 > 1	0.313
Surfactant use	0 (0.0%)	32 (20.9%)	23 (59.0%)	47 (77.0%)	17 (85.0%)	<0.001 ***	3,4,5 > 1,2	0.572

*p* values were calculated by ANOVA test; SD, standard deviation; * *p* < 0.05, ** *p* < 0.01, *** *p* < 0.001; n.s.: non-significant difference. Groups: 1: Full term, 2: Prematurity without ROP, 3: Prematurity with ROP (without Tx), 4: Prematurity with ROP (IVB), 5: Prematurity with ROP (Laser/Laser + IVB). Abbreviations: ASD: atrial septum deficit; BPD: bronchopulmonary dysplasia; BW: birth body weight; NEC: necrotizing enteritis; GA: gestational age; IVB: intravitreal injection of bevacizumab; IVH: intraventricular hemorrhage; PDA: patent ductus arteriosus; PVL: periventricular leukomalacia; RDS: respiratory distress syndrome; ROP: retinopathy of prematurity; Tx: treatment; VSD: ventricular septum deficit. ^a^ Effect size: eta squared for the ANOVA test; Cramer’s V for the X^2^-suqare test.

**Table 2:** WPPSI variables compared between groups after adjusting for age, GA, BPD, and IVH (ANCOVA) (n = 297).

	1. Full-Term (n = 25)	2. Prematurity without ROP (n = 152)	3. Prematurity with ROP (without Tx) (n = 38)	4. Prematurity with ROP (IVB) (n = 62)	5. Prematurity with ROP (Laser/Laser + IVB) (n = 20)	*p* Value	Post Hoc (Bonferroni)	Effect Size ^a^
FSIQ-I	100.7 ± 3.98	95.03 ± 1.27	89.2 ± 2.43	88.71 ± 2.19	85.07 ± 3.7	0.047 *	n.s.	
FSIQ-PR	52.77 ± 7.26	39.72 ± 2.32	31.60 ± 4.43	31.45 ± 3.99	23.93 ± 6.75	0.100	n.s.	
Index scores								
VCI	106.77 ± 4.76	99.19 ± 1.48	94.49 ± 2.92	91.41 ± 2.91	88.1 ± 5.16	0.108	n.s.	
VC-PR	63.21 ± 7.95	48.17 ± 2.49	40.07 ± 4.88	39.46 ± 4.92	27.10 ± 8.63	0.086	n.s.	
VSI	97.76 ± 4.37	95.70 ± 1.37	93.36 ± 2.68	88.71 ± 2.71	86.90 ± 4.75	0.245	n.s.	
VS-PR	46.90 ± 7.68	43.24 ± 2.40	40.22 ± 4.70	36.85 ± 4.69	30.54 ± 8.32	0.666	n.s.	
FRI	109.58 ± 6.48	99.20 ± 2.15	88.55 ± 4.60	82.56 ± 3.78	78.05 ± 5.58	0.004 **	1,2 > 4,5	0.121
FR-PR	65.15 ± 10.93	47.82 ± 3.66	27.57 ± 7.79	23.09 ± 6.49	13.16 ± 9.49	0.008 **	n.s.	
WMI	92.18 ± 3.88	94.42 ± 1.23	87.22 ± 2.36	88.83 ± 2.41	88.87 ± 4.19	0.080	n.s.	
WM-PR	35.18 ± 7.07	40.21 ± 2.23	26.60 ± 4.30	31.37 ± 4.38	29.90 ± 7.63	0.072	n.s.	
PSI	101.24 ± 7.40	89.72 ± 2.37	87.88 ± 5.53	81.14 ± 4.30	79.02 ± 6.68	0.350	n.s.	
PS-PR	49.65 ± 10.81	34.89 ± 3.47	27.26 ± 8.04	21.64 ± 6.46	21.51 ± 9.93	0.405	n.s.	
Subtest scores								
SI	11.25 ± 1.07	9.74 ± 0.39	9.41 ± 0.75	9.32 ± 0.54	9.40 ± 0.79	0.683	n.s.	
IN	10.55 ± 0.78	9.40 ± 0.25	9.04 ± 0.49	9.17 ± 0.44	7.86 ± 0.75	0.249	n.s.	
OA	10.24 ± 0.85	9.23 ± 0.27	8.81 ± 0.53	7.79 ± 0.48	6.46 ± 0.90	0.034 *	1,2 > 5	0.037
BD	8.81 ± 0.90	9.19 ± 0.29	8.50 ± 0.54	8.02 ± 0.49	7.59 ± 0.83	0.275	n.s.	
PC	10.75 ± 1.36	9.95 ± 0.45	8.40 ± 0.94	7.35 ± 0.80	5.71 ± 1.22	0.034 *	1,2 > 5	0.087
MR	10.26 ± 0.82	9.35 ± 0.26	8.15 ± 0.50	8.34 ± 0.45	8.43 ± 0.79	0.190	n.s.	
ZL	7.76 ± 0.71	9.26 ± 0.22	8.29 ± 0.44	8.12 ± 0.43	8.28 ± 0.73	0.014 *	n.s.	
PM	9.33 ± 0.83	8.75 ± 0.26	7.43 ± 0.51	8.11 ± 0.52	8.30 ± 0.90	0.255	n.s.	
CA	9.77 ± 1.21	9.04 ± 0.40	7.57 ± 0.84	6.96 ± 0.71	7.19 ± 1.03	0.230	n.s.	
BS	11.76 ± 1.39	8.44 ± 0.46	7.12 ± 0.97	5.93 ± 0.82	4.47 ± 1.22	0.020 *	1 > 4,5	0.095

Abbreviations: GA: gestational age; BPD: bronchopulmonary dysplasia; IVH: intraventricular hemorrhage; FSIQ-I: Full-Scale Intelligence Quotient index; PR: percentile ranking; VCI: Verbal Comprehension Index; VSI: Visual Spatial Index; FRI: Fluid Reasoning Index; WMI: Working Memory Index; PSI: Processing Speed Index; SI: Similarity; IN: Information; OA: Object Assembly; BD: Block Design; PC: Picture Concept; MR: Matric Reasoning; ZL: Zoo Locations; PM: Picture Memory; CA: Cancellation; BS: Bug Search. * *p* < 0.05, ** *p* < 0.01; n.s.: non-significant difference. ^a^ Effect size: eta squared.

**Table 3:** Visual parameters between groups (ANOVA) (n = 142).

	1. Full-Term (n = 16)	2. Prematurity without ROP (n = 75)	3. Prematurity with ROP (without Tx) (n = 11)	4. Prematurity with ROP (IVB) (n = 27)	5. Prematurity with ROP (Laser/Laser + IVB) (n = 13)	*p* Value	Post Hoc (Bonferroni)	Effect Size ^a^
Uncorrected VA	0.17 ± 0.16	0.23 ± 0.25	0.23 ± 0.14	0.3 ± 0.27	0.64 ± 0.42	<0.001 ***	5 > 1,2,3,4	0.182
BCVA	0.05 ± 0.09	0.1 ± 0.14	0.08 ± 0.06	0.09 ± 0.1	0.14 ± 0.15	0.384	n.s.	
SPH	1.02 ± 1.43	0.85 ± 1.25	0.5 ± 0.68	1.07 ± 2.29	−1.62 ± 3.16	<0.001 ***	1,2,3,4 > 5	0.167
CYL	−1.13 ± 0.63	−4.17 ± 16.47	−1.21 ± 1.1	−1.13 ± 0.99	−1.9 ± 0.65	0.865	n.s.	
SE	1 ± 1.89	−1.09 ± 8.6	−0.36 ± 1.18	1.1 ± 1.35	−3.18 ± 3.4	0.416	n.s.	

Abbreviations: BCVA: best corrected visual acuity; CYL: cylindricity; SPH: spherical correction; SE: spherical equivalent; VA: visual acuity. *** *p* < 0.001; n.s.: non-significant difference. Note: Only visual data from right eye (OD) were used. The VA is presented by logMAR values, with smaller values indicating better vision. ^a^ Effect size: eta squared.

**Table 4:** (**a**) Pearson’s correlation between WPPSI parameters and visual outcomes across five groups (n = 142). (**b**) Pearson’s correlation between WPPSI-IV parameters and visual outcomes across five groups after adjusting for age, GA, BPD, and IVH (n = 142).

(a)				
	BCVA	SPH	CYL	SE
Age	−0.207 *	−0.230 **	−0.037	−0.141
Gender	0.054	0.084	−0.147	−0.079
GA	−0.230 **	0.139	−0.079	−0.012 **
BW	−0.200 *	0.148	−0.095	−0.010
FSIQ-I	−0.173 *	−0.034	0.095	0.086
FSIQ-PR	−0.183 *	−0.029	0.108	0.096
Index scores				
VCI	−0.176	−0.081	0.062	0.040
VC-PR	−0.291 **	−0.060	0.106	0.090
VSI	−0.109	0.043	−0.105	−0.061
VS-PR	−0.107	0.050	−0.138	−0.088
FRI	−0.098	0.039	0.057	0.063
FR-PR	−0.109	0.055	0.081	0.088
WMI	−0.011	0.058	0.158	0.171
WM-PR	−0.019	0.103	0.151	0.177
PSI	−0.158	−0.033	−0.031	−0.029
PS-PR	−0.200	−0.013	0.010	0.020
Subtest scores				
SI	−0.231 *	−0.194	0.000	−0.043
IN	−0.278 **	−0.026	0.136	0.137
OA	−0.055	0.041	−0.181	−0.130
BD	−0.142	0.034	−0.001	0.031
PC	−0.080	0.048	−0.032	−0.012
MR	−0.059	−0.067	0.083	0.041
ZL	−0.141	0.007	0.237 *	0.222
PM	0.133	0.071	0.030	0.058
CA	−0.100	0.033	−0.170	−0.136
BS	−0.317 **	−0.004	0.117	0.120
**(b)**				
**Variable**	**BCVA**	**SPH**	**CYL**	**SE**
FSIQ_I	−0.12	−0.164	0.144	0.094
FSIQ_PR	−0.122	−0.15	0.16	0.11
VCI	−0.192 *	−0.176	0.092	0.05
VCI_PR	−0.286 **	−0.154	0.151	0.115
VSI	−0.056	−0.073	−0.082	−0.087
VSI_PR	−0.045	−0.073	−0.121	−0.12
FRI	0.007	−0.021	0.096	0.087
FRI_PR	0.017	−0.005	0.128	0.12
WMI	0.086	−0.047	0.211	0.168
WMI_PR	0.088	0.017	0.208	0.183
PSI	−0.071	−0.085	−0.006	−0.016
PSI_PR	−0.119	−0.065	0.055	0.05
SI	−0.108	−0.255 *	0.038	−0.009
IN	−0.299 ***	−0.119	0.157	0.127
OA	0.04	−0.032	−0.164	−0.143
BD	−0.083	−0.061	0.027	0.017
PC	0.038	−0.009	0.000	0.004
MR	0.035	−0.121	0.121	0.063
ZL	−0.038	−0.082	0.301 *	0.232
PM	0.21 *	−0.034	0.045	0.028
CA	−0.042	−0.013	−0.15	−0.127
BS	−0.228 *	−0.073	0.161	0.14

Pearson’s correlation coefficient (*r*) analysis. * *p* < 0.05, ** *p* < 0.01, *** *p* < 0.001. Abbreviations: FSIQ-I: Full-Scale Intelligence Quotient index; PR: percentile ranking; VCI: Verbal Comprehension Index; VSI: Visual Spatial Index; FRI: Fluid Reasoning Index; WMI: Working Memory Index; PSI: Processing Speed Index; SI: Similarity; IN: Information; OA: Object Assembly; BD: Block Design; PC: Picture Concept; MR: Matric Reasoning; ZL: Zoo Locations; PM: Picture Memory; CA: Cancellation; BS: Bug Search; BCVA: best corrected visual acuity; CYL: cylindricity; SPH: spherical correction; SE: spherical equivalent; VA: visual acuity. Note: Since VA is presented by logMAR values, hence smaller values indicating better vision, a negative correlation r value means a positive relation. Note: Since VA is presented by logMAR values, hence smaller values indicating better vision, a negative correlation r value means a positive correlation.

## Data Availability

The original contributions presented in the study are included in the article/Supplementary Material, further inquiries can be directed to the corresponding author.

## Supplementary Materials

The following supporting information can be downloaded at: https://www.mdpi.com/article/10.3390/children11080894/s1, Table S1: The difference test of two correlations for each two-group pair.

## Abbreviations

Bayley-III = Bayley Scales of Infant and Toddler Development, Third Edition; BS = bug search; BW = birth weight; ETROP = Early Treatment for Retinopathy of Prematurity; FSIQ = Full-Scale Intelligence Quotient; FRI = fluid reasoning index; GA = gestational age; IVB = intravitreal injection of bevacizumab; IVH = intraventricular hemorrhage; NEC = necrotizing enterocolitis; OA = object assembly; PC = picture concept; PVL = periventricular leukomalacia; ROP = retinopathy of prematurity; VEGF = vascular endothelial growth factor; WPPSI-IV = Wechsler Preschool and Primary Scale of Intelligence, Fourth Edition; ZL = zoo locations
