# Supplementary material for: Relations between Neurocognitive Function and Visual Acuity: A Cross-Sessional Study in a Cohort of Premature Children
Source: Children (Basel). 2024 Jul 25;11(8):894. doi: 10.3390/children11080894 (PMC11352678; doi:10.3390/children11080894)
Supplement: Supplementary file 1 [file children-11-00894-s001.zip › children-3074540-SI.pdf]

Table S1. The difference test of two correlations for each two-group pair

Variables	Comparing group	Correlation of the first group	Correlation of the second group	Difference of correlation	Fisher's z	p
BCVA and BD	1 and 4	-0.607	0.355	-0.962	-2.973	0.003
BCVA and BS	1 and 3	-0.082	0.957	-1.039	-2.989	0.003
BCVA and BS	2 and 3	-0.264	0.957	-1.221	-3.664	<0.001
BCVA and BS	3 and 4	0.957	-0.107	1.065	3.127	0.002
BCVA and BS	3 and 5	0.957	-0.128	1.085	2.955	0.003
SPH and VSI_PR	2 and 4	0.011	-0.700	0.711	3.128	0.002
SPH and VSI_PR	4 and 5	-0.7	0.527	-1.227	-3.206	0.001
SPH and BD	1 and 3	0.442	-0.764	1.206	3.245	0.001
SPH and ZL	1 and 5	-0.032	0.928	-0.960	-3.354	0.001
SPH and ZL	2 and 5	-0.043	0.928	-0.971	-3.939	<0.001
SPH and ZL	4 and 5	-0.429	0.928	-1.357	-4.394	<0.001
CYL and FSIQ_I	1 and 2	0.948	0.183	0.765	3.073	0.002
CYL and FSIQ_I	1 and 3	0.948	-0.330	1.278	3.051	0.002
CYL and FSIQ_I	1 and 4	0.948	-0.249	1.198	3.677	<0.001
CYL and FSIQ_I	1 and 5	0.948	-0.499	1.447	3.769	<0.001
CYL and FSIQ_PR	1 and 4	0.908	-0.261	1.169	3.173	0.002
CYL and FSIQ_PR	1 and 5	0.908	-0.575	1.483	3.467	0.001
CYL and VSI	1 and 2	0.913	-0.143	1.056	3.177	0.001
CYL and VSI	1 and 4	0.913	-0.206	1.119	3.036	0.002
CYL and FRI_PR	2 and 4	0.15	-0.809	0.96	3.021	0.003
CYL and WMI	2 and 3	0.312	-0.937	1.25	3.837	<0.001
CYL and WMI	3 and 4	-0.937	-0.061	-0.876	-2.865	0.004
CYL and WMI_PR	2 and 3	0.290	-0.898	1.188	3.316	0.001
CYL and PSI_PR	1 and 5	0.903	-0.886	1.789	3.340	0.001
CYL and IN	1 and 3	-0.568	-1.000	0.432	6.467	<0.001
CYL and IN	2 and 3	0.209	-1.000	1.209	10.219	<0.001
CYL and IN	3 and 4	-1.000	-0.404	-0.596	-8.509	<0.001
CYL and IN	3 and 5	-1.000	-0.721	-0.278	-6.87	<0.001
CYL and BD	1 and 4	0.876	-0.406	1.282	3.182	0.001
CYL and BD	1 and 5	0.876	-0.471	1.348	2.986	0.003
CYL and MR	1 and 2	0.955	0.158	0.797	3.249	0.001
CYL and MR	1 and 4	0.955	-0.264	1.219	3.831	<0.001
CYL and MR	1 and 5	0.955	-0.303	1.258	3.407	0.001
CYL and MR	2 and 3	0.158	0.993	-0.835	-5.072	<0.001
CYL and MR	3 and 4	0.993	-0.264	1.257	5.552	<0.001
CYL and MR	3 and 5	0.993	-0.303	1.297	4.907	<0.001
CYL and BS	1 and 2	0.998	0.199	0.799	4.582	<0.001
CYL and BS	1 and 3	0.998	-0.198	1.196	3.756	<0.001
CYL and BS	1 and 4	0.998	-0.452	1.45	5.042	<0.001
CYL and BS	1 and 5	0.998	0.002	0.997	4.353	<0.001
SE and WMI_PR	1 and 5	-0.363	0.930	-1.293	-3.036	0.002

SE and WMI_PR	2 and 5	0.27	0.930	-0.659	-2.86	0.004
SE and WMI_PR	3 and 5	-0.328	0.930	-1.258	-2.977	0.003
SE and BD	1 and 3	0.678	0.999	-0.321	-4.008	<0.001
SE and BD	2 and 3	0.022	0.999	-0.977	-6.86	<0.001
SE and BD	3 and 4	0.999	-0.074	1.073	6.635	<0.001
SE and BD	3 and 5	0.999	-0.584	1.582	6.904	<0.001
SE and ZL	1 and 2	-0.81	0.379	-1.189	-2.872	0.004
SE and ZL	1 and 5	-0.81	0.912	-1.722	-3.971	<0.001
SE and ZL	4 and 5	-0.069	0.912	-0.981	-3.017	0.003
SE and CA	2 and 4	-0.244	0.759	-1.004	-2.944	0.003
SE and BS	1 and 2	0.988	0.162	0.826	3.265	0.001
SE and BS	1 and 3	0.988	-0.285	1.273	2.847	0.004
SE and BS	1 and 4	0.988	0.125	0.863	3.028	0.002
SE and BS	1 and 5	0.988	-0.121	1.109	3.277	0.001

Abbreviations: BCVA: best corrected visual acuity; CYL: cylindricity; SPH: spherical correction; SE: spherical equivalent; FSIQ-I: Full-Scale Intelligence Quotient index; PR: percentile ranking; VCI: Verbal Comprehension Index; VSI: Visual Spatial Index; FRI: Fluid Reasoning Index; WMI: Working Memory Index; PSI: Processing Speed Index; SI: Similarity; IN: Information; OA: Object Assembly; BD: Block Design; PC: Picture Concept; MR: Matric Reasoning; ZL: Zoo Locations; PM: Picture Memory; CA: Cancellation; BS: Bug Search;
1: Full term; 2: Prematurity without ROP; 3: Prematurity with ROP (without Tx);
4: Prematurity with ROP (IVI); 5: Prematurity with ROP (Laser/Laser + IVI)
